# A pulmonary artery was embolized in a patient with an occluded pulmonary vein to manage massive hemoptysis

**DOI:** 10.1186/s12890-024-02968-0

**Published:** 2024-04-22

**Authors:** Dongping Xia, Wenhao Cao, Yi Hu

**Affiliations:** 1 Department of Respiratory and Critical Care Medicine, The Central Hospital of Wuhan, Tongji Medical College, Huazhong University of Science and Technology, Wuhan, China; 2 Wuhan Clinical Research Center for Interventional Diagnosis and Treatment of Respiratory Diseases, Wuhan, China

## Abstract

**Background:**

Stenosis and obliteration of the pulmonary vein can be developed by multiple diseases and might cause hemoptysis. Traditional therapy including surgical procedure and conservative treatments might be inappropriate choices to manage massive hemoptysis.

**Case presentation:**

A 64-year-old man, diagnosed with advanced stage IVA lung squamous cell carcinoma, presented with dyspnea and recurrent, massive hemoptysis. An initial contrast-enhanced computed tomography revealed a giant tumor in the left lung hilus and occlusion of the left superior pulmonary vein. Despite immediate selective bronchial artery embolization and simultaneous embolization of an anomalous branch of the internal thoracic artery, the massive hemoptysis continued. Subsequently, embolization of the left superior pulmonary artery was performed, achieving functional pulmonary lobectomy, which successfully treated the hemoptysis without relapse during a six-month follow-up. The patient continues to undergo cancer therapy and remains stable.

**Conclusions:**

This case successfully managed massive hemoptysis associated with lung cancer invasion into the pulmonary vein through functional pulmonary lobectomy via embolization of the corresponding pulmonary artery.

**Supplementary Information:**

The online version contains supplementary material available at 10.1186/s12890-024-02968-0.

## Background

Stenosis and obliteration of the pulmonary vein can be developed by multiple diseases, including ablation for atrial fibrillation, pulmonary vein leiomyosarcoma and uncommon invasion of lung cancer [1, 2]. In these cases, hemoptysis may present as one of those severe complications due to the hemorrhage caused by rupture of pulmonary capillary associated with segmental pulmonary venous hypertension [3]. Under certain circumstances, surgical procedure and conservative treatments might be inappropriate choices to manage massive hemoptysis, and bronchoscopic management was intolerant for the patient [2]. Here we report an unusual interventional therapy of pulmonary artery in managing massive hemoptysis caused by obliteration of the pulmonary vein in a patient with lung cancer.

## Case presentation

**Fig. 1:**
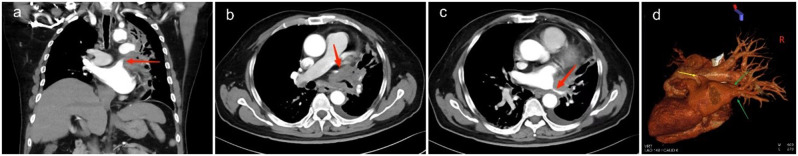
(**a**) Coronal contrast-enhanced computed tomography (CT) of the chest demonstrating obliteration of the left superior pulmonary vein resulting from invasion of tumor (arrow). (**b**) Axial CT shows the obliteration of the left superior pulmonary vein (arrow). (**c**) Axial CT shows the left inferior pulmonary vein stenosis (arrow). (**d**) CT three-dimensional reconstructed image shows the obliteration of the left superior pulmonary vein (yellow arrow) and normal right pulmonary vein (green arrow)

The patient, a 64-year-old male with a history of lifelong smoking and underlying conditions of bronchiectasis, hypertension, and diabetes mellitus, experienced recurrent and massive hemoptysis lasting six days (once or twice daily), with approximately 200 ml of bright red blood per episode. Prior to admission, he had received two courses of chemotherapy combined with immunotherapy for advanced lung squamous cell carcinoma (stage IVA). Physical examination revealed decreased breath sounds on the left side. The patient was hypoxic, requiring nasal cannula oxygenation at 3 L/min (oxygen saturation was 99%), and exhibited elevated leucocyte (22.2 × 10^9/L), neutrophil counts (19.6 × 10^9/L), and high sensitivity C-reactive protein (16.20 mg/L). A contrast-enhanced computed tomography (CT) showed a giant tumor (45 mm minor axis diameter) in the left lung hilus and occlusion of the left superior pulmonary vein (Fig. 1), and the left superior pulmonary artery in the tumor (Figure S1 in the supplementary data online). Destroyed lung associated with bronchiectasis can be located at the upper lobe of left lung (Figure S2 in the supplementary data).

**Fig. 2:**
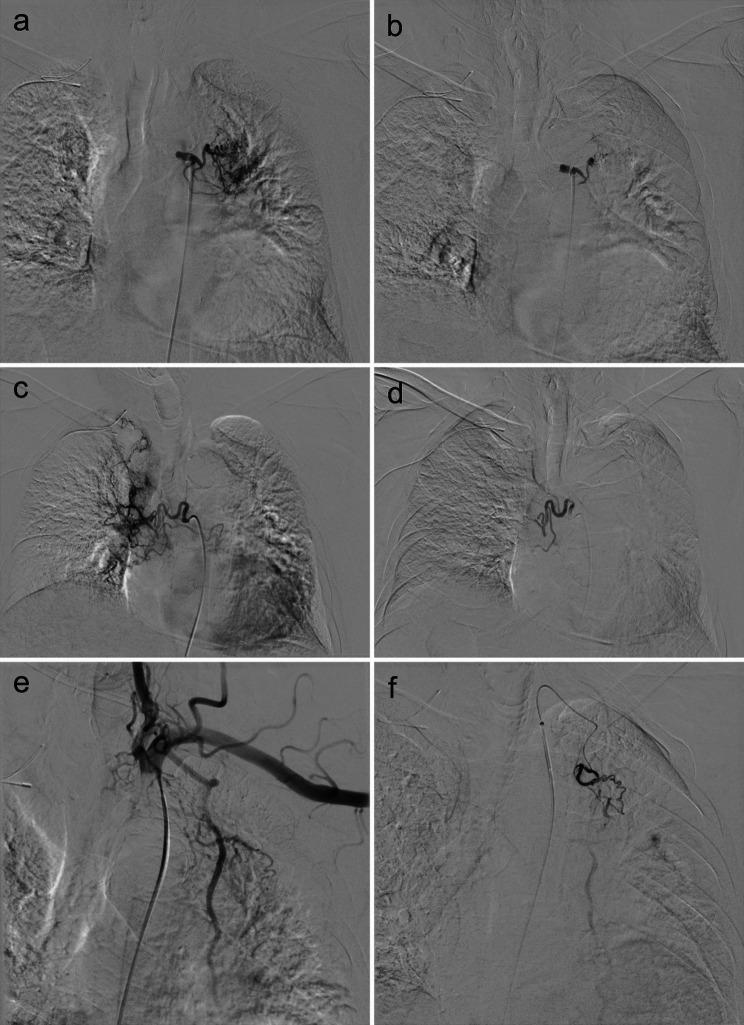
(**a**) Angiographic image shows the branches of the left bronchial artery were tortuous and dilated. (**b**) The left bronchial artery embolization was performed. (**c**) The branches of the right bronchial artery were tortuous and dilated. (**d**) The right bronchial artery embolization was performed. (**e**) The anomalous branches of the left internal thoracic artery were tortuous and dilated. (**f**) The left internal thoracic artery embolization was performed simultaneously

**Fig. 3:**
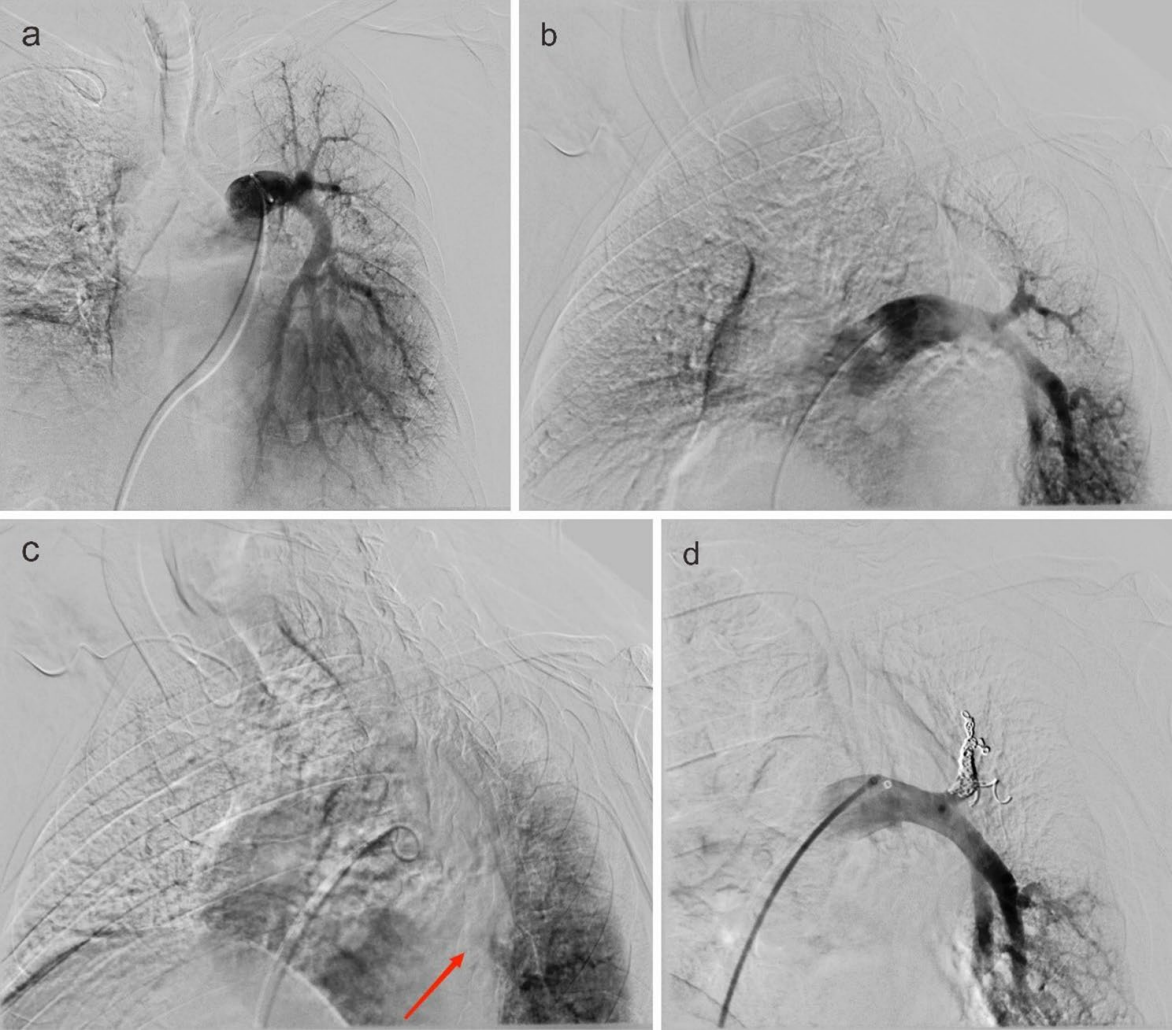
(**a**, **b**) Anteroposterior and lateral view of left main pulmonary artery angiographic image (before the embolization of left upper pulmonary artery) clearly shows the left inferior peripheral pulmonary artery, but unclearly shows the left superior peripheral pulmonary artery. (**c**) Later period of the lateral view of left main pulmonary artery angiographic image (before the embolization of left upper pulmonary artery) shows the left inferior pulmonary vein (red arrow), but not the left superior pulmonary vein. (**d**) The lateral view of left main pulmonary artery angiographic image (after the embolization of left upper pulmonary artery) shows good blood filing in the left inferior pulmonary artery while no blood filling in the left upper pulmonary artery

To address the causative artery based on angiography, selective bilateral bronchial artery embolization was promptly performed after necessary preparations, and concurrent embolization of an anomalous internal thoracic artery branch was also finished (Fig. 2). However, this intervention did not mitigate the hemoptysis. Given the patient’s ineligibility for surgical operation, the identification of the left superior pulmonary vein occlusion as the hemoptysis source, and the fact that the upper lobe of left lung was destroyed and non-functional, embolization of the left superior pulmonary artery was elected to effectuate a functional pulmonary lobectomy (Fig. 3). Besides, the patient declined to undergo a bronchoscopy. Fortunately, this strategy proved successful, with the patient’s hemoptysis resolving completely over a six-month follow-up period, and no pulmonary infarction was detected on the CT reexamination.

## Discussion and conclusion

Pulmonary vein stenosis, though a rare complication of atrial fibrillation ablation and cancer which can be treated by balloon angioplasty and stenting in some cases [1, 4], can lead to severe stenosis and eventual venous occlusion, causing multiple severe complications including hemoptysis.

Pulmonary vein occlusion has been assessed to be associated with various conditions, including radiotherapy and systemic sclerosis, and occurs in a subset of advanced lung cancer cases [2]. Although uncommon, the invasion of tumor into pulmonary vein have been reported previously [5]. Traditional treatment options like pneumonectomy and palliative chemotherapy have limitations. Considering that our patient was experiencing massive hemoptysis and total occlusion precluded the undergoing of surgery or other conservative therapy, we chose the interventional therapy to embolize the culprit vessel. After the angiography, we first empirically performed the bronchial artery embolization but failed to stop the hemoptysis. Then the occlusion of left superior pulmonary vein which is related to the development of pulmonary infarction manifesting as hemoptysis raises concern. Besides, the terminated blood flow of the pulmonary circuit in the left upper lung might cause pulmonary congestion which also could contribute to the massive hemoptysis. Moreover, the upper lobe of the left lung was destroyed before admission. Thus, the decision was made to embolize the left upper pulmonary artery, achieving effective hemostasis without recurrence.

There are no guidelines in performing pulmonary artery embolization. Marcelin and colleagues evaluated the safety and efficacy of endovascular management of pulmonary artery lesions caused by lung tumors, including the irregularity of pulmonary arterial wall and pseudoaneurysm, and claimed that embolization is effective and safe to manage the pulmonary arterial bleeding [6]. However, our case marks the first instance of successfully managing massive hemoptysis associated with lung cancer invasion into the pulmonary vein through functional pulmonary lobectomy via embolization of the corresponding pulmonary artery. This approach may offer an alternative solution for patients who are ineligible for surgery in cases of pulmonary vein occlusion.

### Electronic supplementary material

Below is the link to the electronic supplementary material.

Supplementary Material 1

## Data Availability

The datasets generated during and/or analyzed during the current study are available from the corresponding author on reasonable request.
