# Supplementary material for: A pulmonary artery was embolized in a patient with an occluded pulmonary vein to manage massive hemoptysis
Source: BMC Pulm Med. 2024 Apr 22;24:192. doi: 10.1186/s12890-024-02968-0 (PMC11034142; doi:10.1186/s12890-024-02968-0)
Supplement: Supplementary file 1 — Supplementary Material 1 [file 12890_2024_2968_MOESM1_ESM.docx]

Figure S1. The infiltration of left superior pulmonary artery from the tumor
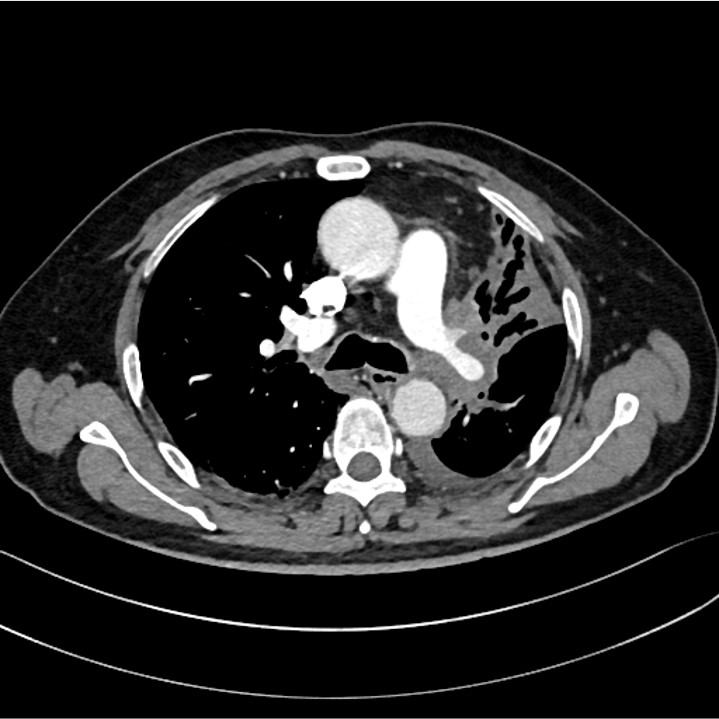


A contrast-enhanced computed tomography (CT) showed a giant tumor and the left superior pulmonary artery infiltration into the tumor

Figure S2. Destroyed lung at the upper lobe of left lung


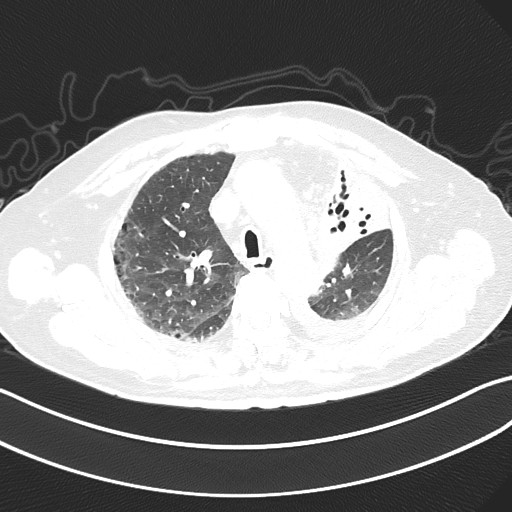


Destroyed lung which might be associated with underlying bronchiectasis can be located at the upper lobe of left lung
